# LRRK2 modulates neuronal vesicles cycle through protein interactions

**DOI:** 10.1186/2193-1801-4-S1-P35

**Published:** 2015-06-12

**Authors:** Giovanni Piccoli, Francesca Pischedda, Daniela Cirnaru, Elisa Greggio, Franco Onofri, Isabella Russo, Antonella Marte, Elisa Belluzzi, Michele Morari, Christian Joannes Gloeckner, Carla Perego, Silvia Marsicano

**Affiliations:** Università Vita-Salute San Raffaele, Italy; Università di Padova, Italy; Università di Genova, Italy; Università degli studi di Milano, Italy; DZNE Tubingen, Germany; Università di Ferrara, Italy

**Keywords:** LRRK2, synaptic vesicle, Parkinson’s disease

Mutations in Leucine-rich repeat kinase 2 gene (LRRK2) are associated with familial and sporadic Parkinson’s disease (PD). LRRK2 is a complex protein that consists of multiple domains executing several functions, including GTP hydrolysis, kinase activity and a C-terminal WD40 domain devoted to protein binding. Robust evidence suggests that LRRK2 acts at the synaptic site as a molecular hub connecting synaptic vesicles to cytoskeletal elements via a complex panel of protein-protein interactions. We have investigated the impact of pharmacological inhibition of LRRK2 kinase activity on synaptic function. Acute treatment with LRRK2 inhibitors reduced the frequency of spontaneous currents, the rate of synaptic vesicle trafficking and the release of neurotransmitter from isolated synaptosomes. The investigation of complementary models lacking LRRK2 expression allowed us to exclude potential off-side effects of kinase inhibitors on synaptic functions. Next we studied whether kinase inhibition affects LRRK2 heterologous interactions. We found that the binding among LRRK2, presynaptic proteins and synaptic vesicles is affected by kinase inhibition. Interestingly, a sequence variant (G2385R) within the WD40 domain has been implicated as a risk factor in PD, but its physiological and pathological function has not been systematically addressed yet. We analyzed molecular features of the WD40 domain and we addressed the functional implication of the G2385R variant. Our results suggest that LRRK2 WD40 domain serves as a hub for protein interactions setting LRRK2 as part of a protein network involved in synaptic vesicle trafficking. Furthermore we showed that the G2385R mutation influences WD40 domain features in terms of domain folding and binding properties and has an impact on synaptic vesicle dynamic. Our results suggest that different PD mutation might influence synaptic vesicle release via modulation of LRRK2 macro-molecular complex.

